# CSF neurofilament light chain predicts 10-year clinical and radiologic worsening in multiple sclerosis

**DOI:** 10.1177/20552173211060337

**Published:** 2021-12-06

**Authors:** Alok Bhan, Cecilie Jacobsen, Ingvild Dalen, Niels Bergsland, Robert Zivadinov, Guido Alves, Kjell-Morten Myhr, Elisabeth Farbu

**Affiliations:** Neuroscience Research Group, Department of Neurology, 60496 Stavanger University Hospital, Stavanger, Norway; Department of Clinical Medicine, 60518 University of Bergen, Bergen, Norway; Neuroscience Research Group, Department of Neurology, 60496 Stavanger University Hospital, Stavanger, Norway; Section of Biostatistics, Department of Research, Stavanger University Hospital, Stavanger, Norway; Buffalo Neuroimaging Analysis Center, Department of Neurology, Jacobs School of Medicine and Biomedical Sciences, University at Buffalo, The State University of New York, Buffalo, NY, USA; IRCCS, Fondazione Don Carlo Gnocchi ONLUS, Milan, Italy; Buffalo Neuroimaging Analysis Center, Department of Neurology, Jacobs School of Medicine and Biomedical Sciences, University at Buffalo, The State University of New York, Buffalo, NY, USA; Center for Biomedical Imaging at Clinical Translational Science Institute, The State University of New York, Buffalo, NY, USA; Neuroscience Research Group, Department of Neurology, 60496 Stavanger University Hospital, Stavanger, Norway; Department of Clinical Medicine, 60518 University of Bergen, Bergen, Norway; Neuro-SysMed – Centre of Excellence for Experimental Therapy in Neurology, Department of Neurology, 60498 Haukeland University Hospital, Bergen, Norway; Neuroscience Research Group, Department of Neurology, 60496 Stavanger University Hospital, Stavanger, Norway; Department of Clinical Medicine, 60518 University of Bergen, Bergen, Norway

**Keywords:** Multiple sclerosis, MRI, biomarkers, CSF, neurofilaments, prognosis, long-term, longitudinal

## Abstract

**Background:**

Neurofilament light chain (NfL) is an attractive biomarker of disease activity and progression in MS, but there is a lack in long-term prognostic data.

**Objective:**

To test the long-term clinical and radiological prognostic value of cerebrospinal fluid (CSF)-NfL among newly diagnosed patients with MS.

**Methods:**

Newly diagnosed MS patients where followed prospectively with baseline CSF-NfL and repeated MRI and clinical assessments for up to 10 years. Associations between baseline CSF-NfL and longitudinal MRI and clinical assessments were found by Generalized Estimating Equations analysis.

**Results:**

Forty-two participants were included. CSF-NfL at baseline was significantly associated with the rate of atrophy in globus pallidus (*p* = 0.009) and hippocampus (*p* = 0.001) as evaluated by MRI. Baseline volumes of thalamus (β −0.33; 95% CI −0.57 to −0.10, *p* = 0.006), T1 (β 0.28; 95% CI 0.11 to 0.44, *p* = 0.001) and T2 (β 0.16; 95% CI 0.04 to 0.27, *p* = 0.008) lesions and baseline levels of CSF-NfL (β 0.9; 95% CI 0.3 to 1.5, *p* = 0.002) significantly predicted EDSS worsening over 10 years. Baseline CSF-NfL gave a comparable prediction to the best MRI volumetric predictors.

**Conclusion:**

CSF-NfL predicted the clinical and radiological course of newly diagnosed patients with MS over a 10-year period, underlining its prognostic role.

## Introduction

Multiple sclerosis (MS) is the leading inflammatory disorder of the central nervous system (CNS) among young adults worldwide, with an escalating prevalence.^1^ Age of onset is about 30 years, and consequently large parts of patients’ lives are affected by the disease. The lack of reliable biomarkers for long-term disease prognosis, combined with a highly heterogeneous disease course, often makes it a life of uncertainty.^2^

At present, magnetic resonance imaging (MRI) is the biomarker of choice for diagnosis and follow-up in MS, and the current McDonald diagnostic criteria are heavily based on MRI.^3,4^ However, there are a number of potential limitations such as time delays from disease onset to image examination, as well as logistics and examination costs. In addition, the limited correlation between traditional MRI measurements and clinical disability impedes the value of MRI as a surrogate marker for MS disease activity.^5,6^ Gadolinium accumulation within the CNS and potential toxicity, which has led to FDA warnings, should also be taken into account.^7,8^ These limitations illustrate an unmet need in the handling of MS which has led to the implementation of composite surveillance strategies, including both MRI and clinical scorings for evaluating disease activity.^9^

Some of these challenges could be solved by implementing new and robust biomarkers. In particular, neurofilaments have emerged as an attractive biomarker of MS disease activity. This group of biomarkers consist of polypeptides found in the cytoplasm of neurons, which are released into the cerebrospinal fluid (CSF) and blood in numerous CNS diseases, including MS.^10^ We and others have previously shown that neurofilament light chain measurements in CSF (CSF-NfL) correlate well with clinical worsening of disability.^11–13^ In addition, both cross-sectional and longitudinal studies report associations of CSF-NfL with MRI activity in MS, but the number of studies are limited, with relatively short follow-up.^14–17^

In this prospective study, we aimed to evaluate the prognostic value of CSF-NfL among newly diagnosed MS-patients in terms of both clinical and radiological disease worsening over a 10-year follow-up.

## Materials and methods

### Patient sample and clinical assessment

This study is part of a prospective population-based longitudinal cohort study of patients with newly diagnosed MS at two specialized centers in South-Western Norway (Stavanger University Hospital in Stavanger and Haukeland University Hospital in Bergen). In total, 108 patients received the diagnosis of MS according to the Poser criteria during 1998 to 2000. Of these, 42 patients with relapsing remitting MS (RRMS) or secondary progressive MS (SPMS) were willing to undergo extended MRI examinations and a lumbar puncture, and were thus included in this study. Upon recruitment, the patients provided written informed consent in study participation, and then underwent standardized clinical evaluations including a full neurological examination and Expanded Disability Status Scale (EDSS) scoring. At the subsequent follow-up with MRI examination and clinical scorings after five and 10 years, in total 37 and 25 patients, respectively, were included.

### MRI

MRI scans were performed at baseline and after 5, and 10 years of follow-up using the same standardized study protocol at both centers. Scans were performed using the same 1.5 T unit (Siemens, Symphony/Philips Medical Systems, Intera). The MRI protocol consisted of dual spin echo (SE) PD/T2-WI, a three-dimensional (3D) T1-W1 and a SE T1-WI. The voxel size for (SE) PD/T2-WI was 0.9  ×  0.9  ×  5.0 mm^3^, for 3D T1-WI 0.9  ×  0.9  ×  1.4 mm^3^, and for SE T1 0.9  ×  0.9  × 5.0 mm^3^ on the Siemens scanner. On the Philips scanner the voxel size for (SE) PD/T2-WI was 0.89  ×  0.89  ×  5.0 mm^3^, for 3D T1-WI 0.89  ×  0.89  × 1.2 mm^3^, and for SE T1 0.89  ×  0.89  ×  5.0 mm^3^. Further details on the MRI protocol have been described previously.^18^

All baseline and follow-up images for each subject were co-registered to its baseline SE T1 image using a 6° of freedom rigid-body model using FMRIB's Linear Image Registration Tool (FMRIB's FLIRT). All subsequent lesion analyses were done using the co-registered images. T1 and T2 lesion volumes were calculated using a reliable semi-automated edge detection contouring/thresholding technique.^19^ Normalised measures for whole brain volume, grey matter volume, white matter volume, cortical volume and lateral ventricular volume were measured using SIENAX (v2.6) with lesion inpainted 3D-T1 images.^20^

Absolute volumes of the subcortical grey matter structures were also derived from using MRIB's Integrated Registration and Segmentation Tool (FIRST v1.2), a model-based segmentation and registration tool.^21^ Normalized subcortical deep grey matter volumes were estimated by multiplying the estimated volumes from FIRST by the volumetric scaling factor from SIENAX.^18^

### CSF sampling and NfL analysis

CSF was obtained at baseline by lumbar puncture using standardized procedures, aliquoted and kept frozen at −70°C. CSF samples had gone through one freeze-thaw cycle before NfL concentrations were quantified using the commercially available sensitive sandwich enzyme-linked immunosorbent assay (ELISA) kit from UmanDiagnostics AB, according to the manufacturer’s kit instructions, described elsewhere.^22^ Intra-assay coefficients of variation were below 15%, and inter-assay coefficients of variation were below 10%.

### Statistical analysis

Means and standard deviations (SD) are presented for continuous variables that were normally distributed and medians and interquartile ranges (IQR) for variables that were not. Counts and percentages are presented for categorical variables.

We used linear regression to study the association between baseline CSF-NfL-levels and baseline MRI brain volumes. CSF-NfL was log transformed using the natural logarithm (after adding a constant 1), to reduce the potential impact of outlying values. T1 and T2 lesion volumes were right-skewed and were thus square root transformed to improve the symmetry of the residual distributions. Results are presented as point estimates of effect (β) with 95% confidence intervals (CI) and with p-values from Wald tests, with and without adjustment for age at baseline, sex and disease duration. For increased interpretability, we calculated the expected difference in brain volumes between CSF-NfL values at the third vs. the first quartile for the statistically significant results. For transformed outcomes, CI for this difference were percentile intervals based on non-parametric bootstrap with 1000 resamples and with predictions based on medium values or most frequent category for the covariates.

Next, Generalized Estimating Equations (GEE) analysis was used to study the association between baseline CSF-NfL levels and longitudinal MRI brain volumes. All models contain time as a continuous explanatory variable as well as the interaction between time and CSF-NfL to assess effect on slope of brain atrophy. Presented results include for each outcome β, 95% CI and Wald *p*-values for the interaction term and the main effect (interpretable as the predicted association between CSF-NfL and brain volume at baseline). We also calculated the expected additional change in MRI brain volumes per five years for CSF-NfL values at the third vs. the first quartile. The exchangeable working correlation structure was used throughout, and robust (sandwich) estimation of standard errors.

Finally, models for longitudinal measures of EDSS score were fitted using GEE, with individual baseline MRI brain volumes or CSF-NfL as predictors. The models and presentation are similar to the previous analyses. Additionally, for these models we present the Corrected Quasi-likelihood under Independence Model Criterion (QICC), which was used for comparison of models with different predictors.

Due to the large number of comparisons, we used *p* < 0.01 as cut off for statistical significance. All analyses were performed in SPSS version 23 (IBM Corp., Armonk, NY, USA), except the bootstrapping which was performed in R version 4.0.2 (R Core Team, 2020).

## Results

### Demographic and clinical profile

Forty-two patients were included with a mean age at baseline of 41.9 years (interquartile range (IQR) 33.2, 50.0), and 29 (69%) of the participants were female. The median disease duration at baseline was 60 months (IQR 39, 171). Thirty-five (83%) patients were categorized as RRMS at baseline and seven (17%) as SPMS. At the five-year follow-up 37 patients were still included and 25 remained at the 10-year follow-up. The proportion with progressive disease increased throughout the study. Only 17% were started on disease-modifying treatment (DMT) at baseline, which had increased to 68% at 10-year follow-up (Table 1).

**Table 1.** Demographics and clinical characteristics at baseline and follow-up visits. Descriptives are presented as median (interquartile range; IQR) unless otherwise specified. Abbreviations: SD Standard deviation; CSF-NfL Cerebrospinal fluid Neurofilament Light; RRMS Relapsing-remitting multiple sclerosis; SPMS Secondary progressive multiple sclerosis; DMT Disease modifying treatment.

	Baseline *N* = 42	5 years *N* = 37	10 years *N* = 25
Age in years at baseline, mean (SD)	41.9 (9.6)	41.9 (9.9)	40.3 (8.5)
Female, *n* (%)	29 (69)	27 (73)	17 (68)
CSF-NfL at baseline, ng/ml	339 (102, 1246)	310 (107, 1144)	302 (106, 1144)
Disease duration in months at baseline	60 (36, 171)	60 (36, 174)	60 (48, 138)
Months since last attack at baseline	9 (1.5, 17)*	10 (1, 20)*	10 (1, 20)
EDSS	3.5 (2.0, 4.0)	3.5 (2.0, 4.0)	3.0 (2.0, 5.0)
Disease course, *n* (%)			
RRMS	35 (83)	26 (70)	16 (64)
SPMS	7 (17)	11 (30)	9 (36)

Patients on DMT, *n* (%)	7 (17)	18 (49)	17 (68)
Interferons	6	13	9
Glatiramer acetate	1	5	3
Natalizumab			3
Fingolimod			1
Mitoxantrone			1

*1 missing.

### Association between CSF-NfL and brain volumes at baseline

When adjusted for age at baseline, sex and disease duration, higher CSF-NfL concentrations were significantly associated with lower MRI volumes of thalamus (β −1.9; 95% CI −3.0 to −0.7, *p* = 0.002), nucleus accumbens (β −0.17; 95% CI −0.30 to −0.04, *p* = 0.009), and higher square-rooted T1- (β 1.7; 95% CI 0.9 to 2.5, *p* < 0.001) and T2-lesion volumes (β 2.3; 95% CI 1.2 to 3.4, *p* < 0.001). (Table 2) Lower volumes of whole brain, white matter, deep grey matter and putamen were also associated with higher CSF-NfL, but did not reach statistical significance (*p*-values between 0.01 and 0.05). When comparing the third and the first quartile value of CSF-NfL concentrations (i.e. CSF-NfL 1.2 vs. 0.1) we found an expected difference in thalamic volume of −1.42 cm^3^ (95% CI −2.0 to 0.5 cm^3^); in nucleus accumbens volume −0.12 cm^3^ (−0.21 to −0.03 cm^3^); in T1 lesion volume of +3.8 cm^3^ (1.8 to 6.0 cm^3^); and in T2 lesion volume +8.5 cm^3^ (3.9 to 12.5 cm^3^).

**Table 2.** Associations between CSF-NfL and MRI brain volumes at baseline for 42 MS patients. Results from linear regression analyses with log-transformed CSF-NfL as independent variable. All volumes are measured in cubic cm. T1 and T2 lesion volumes were square root transformed. Adjusted models include covariates age at baseline, sex and disease duration. P-values from Wald tests. Statistically significant results (p < 0.01) are printed in boldface. CI, confidence interval; LV, lesion volume; CSF-NfL, Cerebrospinal fluid Neurofilament Light Chain; MRI, Magnetic Resonance Imaging.

	Unadjusted		Adjusted	
Volumetric outcome	β (95% CI)	*p*	β (95% CI)	*p*
Whole brain	−36 (−92, 20)	0.20	−60 (−114, −6)	0.030
White matter	−25 (−50, 1)	0.056	−33 (−61, −4)	0.025
Total grey matter	−11 (−49, 26)	0.55	−27 (−61, 7)	0.11
Ventricle	−2 (−16, 12)	0.79	6 (−8, 19)	0.42
Cortical grey matter	−17 (−49, 14)	0.27	−26 (−56, −4)	0.083
Deep grey matter	−0.3 (−3.8, 3.2)	0.86	−4.3 (−7.6, −0.9)	0.014
Thalamus	−0.7 (−1.8, −0.4)	0.22	−1.8 (−2.9, −0.7)	0.002
Pallidus	−0.02 (−0.32, 0.27)	0.88	−0.26 (−0.58, 0.06)	0.10
Putamen	0.06 (−0.86, 0.97)	0.90	−1.0 (−1.9, −0.2)	0.018
Caudate	0.08 (−0.48, 0.65)	0.77	−0.45 (−1.05, 0.14)	0.13
Hippocampus	0.35 (−0.38, 1.08)	0.34	−0.20 (−0.99, 0.60)	0.62
Amygdala	−0.13 (−0.42, 0.15)	0.35	−0.33 (−0.67, −0.00)	0.049
Nuccleus Accumbens	−0.03 (−0.17, 0.11)	0.69	−0.17 (−0.30, −0.04)	0.009
T1 Lesion	0.9 (0.0, 1.8)	0.046	1.7 (0.9, 2.5)	<0.001
T2 Lesion	1.2 (−0.1, 2.4)	0.068	2.3 (1.2, 3.4)	<0.001

### Association between baseline CSF-NfL and long-term MRI brain atrophy

When adjusting for age at baseline, sex, and disease duration we found significant CSF-NfL-dependent slopes for volumes of globus pallidus (β −0.20; 95% CI −0.35 to −0.05, *p* = 0.009) and hippocampus (β −0.32; 95% CI −0.50 to −0.14, *p* = 0.001), for both of which higher CSF-NfL predicted a higher rate of atrophy (Table 3). When comparing the third vs. the first quartile of CSF-NfL, higher CSF-NfL was associated with an additional volume loss of globus pallidus of 0.14 cm^3^ (95% CI 0.03 to 0.24 cm^3^) per five years, which amounted to 280% of the median change in globus pallidus volume from baseline to five years (Table S1). The additional loss of volume in hippocampus for the third compared to the first quartile was 0.22 cm^3^ (95% CI 0.10 to 0.35 cm^3^), amounting to 275% of the median changes in the volume from baseline to five years.

**Table 3.** Associations between baseline CSF-NfL-levels and longitudinal measures of MRI brain structure volumes for 42 MS patients. Results from Generalized Estimating Equations (GEE) analysis with exchangeable working correlation and with log-transformed CSF-NfL as independent variable (predictor). All models include effects of time (per 5 years) and CSF-NfL, as well as the interaction between time and CSF-NfL. Adjusted models include covariates age at baseline, gender and disease duration. All volumes are measured in cubic cm. T1 and T2-volumes were square root transformed. *P*-values from Wald tests. Statistically significant results (*p* < 0.01) are printed in boldface. Abbreviations: CSF-NfL, Cerebrospinal Fluid Neurofilament Light Chain; MRI, Magnetic Resonance Imaging; No. obs, Number of observations; CI, Confidence Interval; LV, Lesion Volume.

		Unadjusted					Adjusted			
		Main effect		Interaction effect			Main effect		Interaction effect	
Volumetric Outcome	No. obs	β (95% CI)	*p*	β (95% CI)	*p*	No. obs	β (95% CI)	*p*	β (95% CI)	*p*
Whole brain	105	−30 (−76, 17)	0.21	9 (−7, 26)	0.27	105	−58 (−99, −19)	0.003	9 (−7, 26)	0.27
White matter	105	−20 (−39, −2)	0.034	9 (−3, 21)	0.14	105	−31 (−51, −11)	0.002	9 (−3, 22)	0.13
Total grey matter	105	−9 (−43, 24)	0.59	0.0 (−9.5, 9.4)	0.99	105	−27 (−53, 0)	0.051	0.0 (−96, 9.6)	1.00
Ventricle	105	−2 (−14, 11)	0.81	6 (1, 12)	0.02	105	7 (−4, 17)	0.23	6 (1, 12)	0.021
Cortical grey matter	105	−16 (−44, 12)	0.26	2 (−6, 10)	0.61	105	−26 (−49, −2)	0.032	2 (−6, 11)	0.61
Deep grey matter	104	−0.7 (−3.3, 2.0)	0.63	−1.5 (−2.7, −0.3)	0.015	104	−4.6 (−7.8, −1.4)	0.005	−1.6 (−2.8, −0.3)	0.012
Thalamus	104	−0.7 (−1.5, 0.2)	0.115	−0.24 (−0.55, 0.08)	0.143	104	−1.9 (−2.8, −1.0)	<0.001	−0.24 (−0.55, 0.08)	0.136
Pallidus	104	−0.06 (−0.33, 0.21)	0.65	−0.21 (−0.35, −0.06)	0.007	104	−0.31 (−0.61, −0.02)	0.037	−0.20 (−0.35, −0.05)	0.009
Putamen	104	−0.10 (−0.85, 0.66)	0.80	−0.53 (−1.03, −0.03)	0.038	104	−1.2 (−2.0, −0.4)	0.004	−0.52 (−1.02, −0.02)	0.04
Caudate	104	0.06 (−0.37, 0.49)	0.78	−0.11 (−0.29, 0.06)	0.21	104	−0.42 (−0.92, 0.08)	0.100	−0.11 (−0.29, 0.65)	0.22
Hippocampus	104	0.29 (−0.39, 0.97)	0.41	−0.31 (−0.49, −0.12)	0.001	104	−0.25 (−1.09, 0.60)	0.57	−0.32 (−0.50, −0.14)	0.001
Amygdala	104	−0.16 (−0.46, 0.14)	0.29	−0.06 (−0.21, 0.09)	0.42	104	−0.33 (−0.67, 0.01)	0.054	−0.06 (−0.21, 0.09)	0.41
Nucleus Accumbens	104	−0.05 (−0.15, 0.05)	0.36	−0.05 (−0.11, 0.02)	0.18	104	−0.19 (−0.28, −0.09)	<0.001	0.04 (−0.11, 0.03)	0.23
T1 Lesion	103	1.0 (0.1, 1.9)	0.034	0.17 (−0.19, 0.53)	0.35	103	1.8 (1.0, 2.7)	<0.001	0.16 (−0.20, 0.52)	0.38
T2 Lesion	103	1.3 (0.2, 2.5)	0.023	0.26 (−0.14, 0.66)	0.20	103	2.5 (1.5, 3.6)	<0.001	0.25 (−0.14, 0.65)	0.21

### Association of longitudinal EDSS score with baseline CSF-NfL and baseline MRI brain volumes

Adjusted for age at baseline, sex and disease duration, baseline MRI volumes of thalamus (β −0.33; 95% CI −0.57 to −0.10, *p* = 0.006), T1-lesions (β 0.28; 95% CI 0.11 to 0.44, *p* = 0.001), T2-lesions (β 0.16; 95% CI 0.04 to 0.27, *p* = 0.008), and baseline levels of CSF-NfL (β 0.9; 95% CI 0.3 to 1.5, *p* = 0.002) each significantly predicted the rate of EDSS worsening over the 10-year follow-up period (Table 4). Based on the QICC, the baseline MRI volume measure that correlated best to the longitudinal EDSS throughout the 10-year follow-up (i.e. lowest QICC) after adjustment was volume of thalamus (QICC = 302). In comparison, the model comprising levels of CSF-NfL had a similar QICC of 284. Without adjusting for age, sex or disease duration, many of the MRI volumes performed better than CSF-NfL as judged by the QICC.

**Table 4.** Associations between longitudinal EDSS score and baseline MRI brain structure volumes or CSF-NfL. 42 patients included at baseline, 37 and 25 patients at five-year and 10-year follow-up, respectively (118 observations). Results from Generalized Estimating Equations (GEE) analysis with exchangeable working correlation. All models include effects of time (per 5 years) and the stated predictor, as well as the interaction between time and the predictor. Adjusted models include covariates age at baseline, sex and disease duration. QICC for the model with just these adjustment variables and time was 375. All volumes are measured in cubic cm. T1 and T2 lesion volumes were square rooted, and CSF-NfL (ng/ml) was log transformed. *P*-values from Wald tests. Statistically significant results (*p* < 0.01) are printed in boldface. Abbreviations: EDSS, Expanded Disability Status Scale; MRI, Magnetic Resonance Imaging; CSF-NfL, Cerebrospinal Fluid Neurofilament Light Chain; MS, Multiple Sclerosis; CI, Confidence Interval; QICC, Corrected Quasi-likelihood under Independence Model Criterion; LV, Lesion Volume.

	Unadjusted					Adjusted				
	Main effect		Interaction			Main effect		Interaction		
Predictor	β (95% CI)	*p*	β (95% CI)	*p*	QICC	β (95% CI)	*p*	β (95% CI)	*p*	QICC
Whole Brain volume	−0.008 (−0.013, −0.003)	0.001	−0.001 (−0.003, 0.002)	0.74	341	−0.007 (−0.014, −0.001)	0.029	−0.001 (−0.003, 0.002)	0.72	344
White matter volume	−0.016 (−0.026, −0.006)	0.002	−0.000 (−0.008, 0.007)	0.90	357	−0.013 (−0.025, −0.002)	0.013	−0.001 (−0.008, 0.006)	0.88	345
Total grey matter volume	−0.011 (−0.018, −0.004)	0.002	−0.001 (−0.005, 0.003)	0.69	352	−0.008 (−0.020, 0.001)	0.065	−0.001 (−0.005, 0.003)	0.68	357
Ventricle volume	0.026 (0.007, 0.040)	<0.001	0.004 (−0.007, 0.016)	0.43	344	0.026 (0.007, 0.045)	0.009	0.004 (−0.007, 0.016)	0.44	346
Cortical grey matter volume	−0.014 (−0.022, −0.006)	0.001	0.000 (−0.005, 0.005)	0.93	352	−0.012 (−0.023, −0.002)	0.025	0.000 (−0.005, 0.005)	0.93	354
Deep grey matter volume	−0.11 (−0.18, −0.04)	0.002	−0.047 (−0.089, −0.005)	0.025	327	−0.13 (−0.22, −0.04)	0.003	−0.048 (−0.091, −0.006)	0.026	303
Thalamic volume	−0.30 (−0.54, −0.07)	0.012	−0.19 (−0.33, −0.05)	0.009	322	−0.36 (−0.57, −0.14)	0.001	−0.33 (−0.57, −0.10)	0.006	302
Pallidus volume	−0.9 (−1.7, −0.1)	0.035	−0.5 (−1.0, −0.1)	0.078	366	−1.2 (−2.0, −0.4)	0.004	−0.5 (−1.0, −0.0)	0.071	327
Putamen volume	−0.37 (−0.61, −0.12)	0.003	−0.12 (−0.27, 0.03)	0.12	350	−0.44 (−0.80, −0.08)	0.017	−0.13 (−0.28, 0.03)	0.107	331
Caudate colume	−0.59 (−0.99, −0.19)	0.004	−0.24 (−0.49, 0.01)	0.059	338	−0.68 (−1.17, −0.19)	0.006	−0.25 (−0.50, 0.01)	0.056	315
Hippocampus volume	−0.35 (−0.70, −0.01)	0.046	−0.16 (−0.38, 0.06)	0.15	369	−0.36 (−0.84, 0.12)	0.14	−0.17 (−0.39, 0.05)	0.14	351
Amygdala volume	−1.2 (−2.0, −0.3)	0.006	−0.4 (−1.1, 0.3)	0.29	361	−1.1 (−2.0, −0.2)	0.015	−0.4 (−1.1, 0.3)	0.26	340
Accumbens volume	−2.9 (−4.0, −1.5)	<0.001	−0.6 (−1.6, 0.5)	0.28	343	−2.9 (−5.0, −0.8)	0.008	−0.6 (−1.7, 0.5)	0.28	336
T1 Lesion volume	0.34 (0.02, 0.65)	0.039	0.27 (0.11, 0.44)	0.001	327	0.35 (−0.04, 0.74)	0.082	0.28 (0.11, 0.44)	0.001	318
T2 Lesion volume	0.25 (0.04, 0.45)	0.019	0.15 (0.04, 0.27)	0.009	337	0.27 (0.02, 0.51)	0.035	0.16 (0.04, 0.27)	0.008	327
CSF-NfL	0.7 (0.0, 1.4)	0.038	0.9 (0.3, 1.5)	0.002	348	1.4 (0.6, 2.2)	<0.001	0.9 (0.3, 1.5)	0.002	284

## Discussion

In this study we demonstrate significant associations between baseline CSF-NfL and MRI parameters both at baseline and over a long-term follow-up of 10 years. At baseline, CSF-NfL was significantly negatively associated with baseline thalamic and nucleus accumbens volumes. Previous studies have reported heterogenous results on the association between CSF-NfL levels in patients with clinically isolated syndrome and grey matter atrophy.^23,24^ Small sample sizes may have attributed to the discrepant results. Nevertheless, thalamic atrophy has been reported from the earliest disease stages of MS, and with strong clinical correlations.^25,26^ We found increasing CSF-NfL to be associated with increasing baseline T1- and T2-lesion volumes, which is consistent with previous studies.^15,27^

We also found significant associations between baseline CSF-NfL and future loss of grey matter volume structures of globus pallidus and hippocampus, as well as trend (*p* between 0.01 and 0.05) between baseline CSF-NfL and future loss of total deep grey matter and putamen. Patients with baseline CSF-NfL in the third quartile compared to the first quartile had a volume loss of globus pallidus and hippocampus of 2.8 times higher than the median change of the whole study population seen in the first five years. To our knowledge, this study is the first to show an association between CSF-NfL and grey matter volumes in a long-term setting. This observation has direct clinical implications, as deep grey matter loss has been associated with disability accumulation, and involvement of the hippocampus has been found to relate to several cognitive functions, including poor verbal memory performance.^28,29^ CSF-NfL was not associated with future thalamic atrophy over the 10 years of follow-up in our study. Previous studies have found thalamus atrophy to commence early in the disease course.^25^ This might play a role in the lack in association in our patients, as our patients already had a considerably long interval from disease onset to diagnosis.

When we adjusted for the confounders age at baseline, sex and disease duration, we found that baseline CSF-NfL-levels predicted further EDSS worsening throughout the study period better than what baseline MRI volumes did, assessed by GEE models using the QICC. These results highlight the prognostic abilities of CSF-NfL in predicting disease worsening in MS. Interestingly, when models were not corrected for adjustment variables, the model for CSF-NfL did not predict the future EDSS to the same degree as what the better models of MRI volumes did. This change in relative performance when adjusting for confounders suggests that CSF-NfL contains valuable information in regards to the clinical worsening in MS not being detected by MRI. In our model, when adjusted for age, sex and disease duration, adding baseline CSF-NfL gave a better prediction of future EDSS than adding for instance baseline thalamus volume to the model. The ability of CSF-NfL in MS to identify a subgroup of MS patients with disease activity that was not detected on MRI was also shown in a study reporting that more than 12% of the patients had elevated CSF-NfL and clinical worsening without MRI activity.^30^

Our study has some limitations, foremost the limited sample size. In addition, our population of patients were diagnosed with MS according to the more stringent Poser criteria. That probably gave longer disease duration before diagnosis than what is common today, combined with an observation period before the definite diagnosis, likely influenced by the limited treatment options at that time. Our population was also more treatment naïve during the follow-up for this reason, and only 17% received DMT at baseline, a time where high-efficacy treatments were not available. However, we believe these factors also represent a strength of this study, as our treatment naïve patients expose the natural relationship of neurofilaments and disease course. Furthermore, spinal MRI was not performed in this study. This might explain some of the lack of correlation between baseline MRI results and CSF-NfL, and between MRI and clinical worsening. Spinal cord lesions are linked to increased disability in MS, and have been shown to increase sNfL.^31,32^ Although this study was performed at two different centers, we did not find any differences in volume measurements that could be attributed to different MRI machines.

We used ELISA technology to assess levels of CSF-NfL in this study. However today even more sensitive methods for detection of low concentration of NfL like the Simoa technology has made it possible to also analyze blood for NfL (sNfL). In a small study from 2016, there was a correlation between sNfL and white matter volume loss, but not grey matter loss over a median follow-up of 3.6 years.^33^ In contrast, more recently Jakimovski and colleagues showed that baseline sNfL levels were associated with future GM atrophy, including total deep GM volume and specific structures such as thalamus, putamen and globus pallidus for a follow-up time of five years.^34^ In addition, another study reported significant associations between baseline sNfL and subsequent brain atrophy at year 10, although sNfL was not deemed to be a good predictor of long-term disability worsening.^35^ However, only sNfL was analyzed in this study, and CSF-NfL has been reported to be a significantly better predictor of “no evidence of disease activity – third revision” (NEDA-3) than sNfL.^15^ Consequently we find it meaningful to analyze for CSF-NfL at time of diagnoses despite the more readily available analysis for sNfL.

As this study only examined patients at three different time points it is of importance that these visits were carried out during a clinically stable time period, and not examined during a relapse. In total eight patients were included in the study that had an attack within the last two months prior to the baseline visit, as well as one patient with an attack around three months prior to the five-year follow-up. However, we did not observe any significant differences between the groups.

We have previously shown correlation between baseline CSF-NfL and worsening of disability defined by EDSS after five years of follow-up^11^ This study further strengthens the use of CSF-NfL as a prognostic biomarker for clinical and radiological disease worsening in newly diagnosed MS. Although the results from the statistical models used in this paper must be interpreted on a group level, there is now increasing evidence that assessing patients at time of diagnosis for CSF-NfL will provide an indication of future disease burden up to 10 years in advance. This further allows for a better stratification of disease severity, and thus a more personalized approach in choosing treatment for MS.

## Supplemental Material

sj-docx-1-mso-10.1177_20552173211060337 - Supplemental material for CSF neurofilament light chain predicts 10-year clinical and radiologic worsening in multiple sclerosis Click here for additional data file. Supplemental material, sj-docx-1-mso-10.1177_20552173211060337 for CSF neurofilament light chain predicts 10-year clinical and radiologic worsening in multiple sclerosis by Alok Bhan, Cecilie Jacobsen, Ingvild Dalen, Niels Bergsland, Robert Zivadinov, Guido Alves, Kjell-Morten Myhr and Elisabeth Farbu in Multiple Sclerosis Journal – Experimental, Translational and Clinical

sj-doc-2-mso-10.1177_20552173211060337 - Supplemental material for CSF neurofilament light chain predicts 10-year clinical and radiologic worsening in multiple sclerosis Click here for additional data file. Supplemental material, sj-doc-2-mso-10.1177_20552173211060337 for CSF neurofilament light chain predicts 10-year clinical and radiologic worsening in multiple sclerosis by Alok Bhan, Cecilie Jacobsen, Ingvild Dalen, Niels Bergsland, Robert Zivadinov, Guido Alves, Kjell-Morten Myhr and Elisabeth Farbu in Multiple Sclerosis Journal – Experimental, Translational and Clinical
