# Supplementary material for: CSF neurofilament light chain predicts 10-year clinical and radiologic worsening in multiple sclerosis
Source: Mult Scler J Exp Transl Clin. 2021 Dec 6;7(4):20552173211060337. doi: 10.1177/20552173211060337 (PMC8652913; doi:10.1177/20552173211060337)
Supplement: sj-docx-1-mso-10.1177_20552173211060337 - Supplemental material for CSF neurofilament light chain predicts 10-year clinical and radiologic worsening in multiple sclerosis [file sj-docx-1-mso-10.1177_20552173211060337.docx]

| **Volumetric outcome** | **n** | **Baseline to 5 years** |  | **n** | **Baseline to 10 years** |
| --- | --- | --- | --- | --- | --- |
| Whole brain | 37 | -19.5 (-31.8, +1.2) |  | 25 | -34.5 (-56.4, -18.0) |
| White matter | 37 | -5.0 (-14.7, +1.8) |  | 25 | -7.8 (-17.2, +5.0) |
| Total grey matter | 37 | -9.2 (-22.0, +0.5) |  | 25 | -25.5 (-38.6, -20.0) |
| Ventricle | 37 | +7.6 (-5.8, +18.4) |  | 25 | +1.9 (-7.6, +20.3) |
| Cortical grey matter | 37 | -9.9 (-16.6, -4.1) |  | 25 | -20.6 (-33.9, -14.0) |
| Deep grey matter | 37 | -0.91 (-1.96, -0.22) |  | 25 | -1.88 (-3.04, -1.19) |
| Thalamus | 37 | -0.26 (-0.58, -0.03) |  | 25 | -0.59 (-0.86, -0.29) |
| Pallidus | 37 | -0.05 (-0.15, +0.06) |  | 25 | -0.08 (-0.19, 0.00) |
| Putamen | 37 | -0.30 (-0.61, +0.07) |  | 25 | -0.33 (-0.88, -0.18) |
| Caudate | 37 | -0.24 (-0.44, -0.02) |  | 25 | -0.42 (-0.61, -0.28) |
| Hippocampus | 37 | -0.08 (-0.40, +0.18) |  | 25 | -0.36 (-0.63, -0.18) |
| Amygdala | 37 | -0.02 (-0.10, +0.07) |  | 25 | -0.03 (-0.23, +0.09) |
| Nucleus Accumbens | 37 | -0.03 (-0.14, +0.03) |  | 25 | -0.11 (-0.23, +0.02) |
| T1 LV | 37 | +0.28 (-0.08, +2.77) |  | 25 | -0.02 (-0.52, +1.18) |
| T2 LV | 37 | +0.74 (-0.43, +6.12) |  | 25 | -0.57 (-5.50, +0.71) |

Table S1: Changes in MRI volumes. Data given as median (interquartile range). All measures in cm3.
