# Supplementary material for: CSF neurofilament light chain predicts 10-year clinical and radiologic worsening in multiple sclerosis
Source: Mult Scler J Exp Transl Clin. 2021 Dec 6;7(4):20552173211060337. doi: 10.1177/20552173211060337 (PMC8652913; doi:10.1177/20552173211060337)
Supplement: sj-doc-2-mso-10.1177_20552173211060337 - Supplemental material for CSF neurofilament light chain predicts 10-year clinical and radiologic worsening in multiple sclerosis [file sj-doc-2-mso-10.1177_20552173211060337.doc]

MS Journal Appendix for MRI methodology

| Hardware | |
| --- | --- |
| Field strength | 1.5 T |
| Manufacturer | Siemens |
| Model | Symphony |
| Coil type  (e.g. head, surface) | Head coil |
| Number of coil channels | 8 |

| Acquisition sequence | | |
| --- | --- | --- |
| Type  (e.g. FLAIR, DIR, DTI, fMRI) | Dual spin echo (SE) PD/T2 | |
| Acquisition time | 15.22 min | |
| Orientation | Axial | |
| Alignment  (e.g. anterior commissure/poster commissure line) | Anterior commissure/posterior commissure | |
| Voxel size | 0.9 x 0.9 x 5.0 mm3 | |
| TR | 3200 ms | |
| TE | 13/81 ms | |
| TI |  | |
| Flip angle | 180˚ | |
| NEX | 2 | |
| Field of view | 230 mm | |
| Matrix size | 256 × 256 | |
| Parallel imaging | Yes | **No** |
| If used, parallel imaging method:  (e.g. SENSE, GRAPPA) |  | |
| Cardiac gating | Yes | **No** |
| If used, cardiac gating method:  (e.g. PPU or ECG) |  | |
| Contrast enhancement | Yes | **No** |
| If used, provide name of contrast agent, dose and timing of scan post-contrast administration |  | |
| Acquisition sequence | | |
| Type  (e.g. FLAIR, DIR, DTI, fMRI) | 3D T1 | |
| Acquisition time | 9.12 min | |
| Orientation | Sagittal | |
| Alignment  (e.g. anterior commissure/poster commissure line) | Anterior commissure/posterior commissure | |
| Voxel size | 0.9 x 0.9 x 1.4 mm3 | |
| TR | 25 ms | |
| TE | 10 ms | |
| TI |  | |
| Flip angle | 50˚ | |
| NEX | 1 | |
| Field of view | 230 mm | |
| Matrix size | 256 × 256 | |
| Parallel imaging | Yes | **No** |
| If used, parallel imaging method:  (e.g. SENSE, GRAPPA) |  | |
| Cardiac gating | Yes | **No** |
| If used, cardiac gating method:  (e.g. PPU or ECG) |  | |
| Contrast enhancement | Yes | **No** |
| If used, provide name of contrast agent, dose and timing of scan post-contrast administration |  | |

| Acquisition sequence | | |
| --- | --- | --- |
| Type  (e.g. FLAIR, DIR, DTI, fMRI) | SE T1 | |
| Acquisition time | 4.27 min | |
| Orientation | Axial | |
| Alignment  (e.g. anterior commissure/poster commissure line) | Anterior commissure/posterior commissure | |
| Voxel size | 0.9 x 0.9 x 5.0 mm3 | |
| TR | 657 ms | |
| TE | 15 ms | |
| TI |  | |
| Flip angle | 90˚ | |
| NEX | 2 | |
| Field of view | 230 mm | |
| Matrix size | 256 × 256 | |
| Parallel imaging | Yes | **No** |
| If used, parallel imaging method:  (e.g. SENSE, GRAPPA) |  | |
| Cardiac gating | Yes | **No** |
| If used, cardiac gating method:  (e.g. PPU or ECG) |  | |
| Contrast enhancement | Yes | **No** |
| If used, provide name of contrast agent, dose and timing of scan post-contrast administration |  | |
| Other parameters: |  | |

| Hardware | |
| --- | --- |
| Field strength | 1.5 T |
| Manufacturer | Philips Medical systems |
| Model | Intera |
| Coil type  (e.g. head, surface) | Head coil |
| Number of coil channels | 7 |

| Acquisition sequence | | |
| --- | --- | --- |
| Type  (e.g. FLAIR, DIR, DTI, fMRI) | Dual spin echo (SE) PD/T2 | |
| Acquisition time | 11.46 min | |
| Orientation | Axial | |
| Alignment  (e.g. anterior commissure/poster commissure line) | Anterior commissure/posterior commissure | |
| Voxel size | 0.89 x 0.89 x 5.0 mm3 | |
| TR | 3240 ms | |
| TE | 19/80 ms | |
| TI |  | |
| Flip angle | 90˚ | |
| NEX | 2 | |
| Field of view | 230 mm | |
| Matrix size | 256 × 256 | |
| Parallel imaging | Yes | **No** |
| If used, parallel imaging method:  (e.g. SENSE, GRAPPA) |  | |
| Cardiac gating | Yes | **No** |
| If used, cardiac gating method:  (e.g. PPU or ECG) |  | |
| Contrast enhancement | Yes | **No** |
| If used, provide name of contrast agent, dose and timing of scan post-contrast administration |  | |

| Acquisition sequence | | |
| --- | --- | --- |
| Type  (e.g. FLAIR, DIR, DTI, fMRI) | 3D T1 | |
| Acquisition time | 8.04 | |
| Orientation | Axial | |
| Alignment  (e.g. anterior commissure/poster commissure line) | Anterior commissure/posterior commissure | |
| Voxel size | 0.89 x 0.89 x 1.2 mm3 | |
| TR | 17 ms | |
| TE | 4 ms | |
| TI |  | |
| Flip angle | 12˚ | |
| NEX | 1 | |
| Field of view | 230 mm | |
| Matrix size | 256 × 256 | |
| Parallel imaging | Yes | **No** |
| If used, parallel imaging method:  (e.g. SENSE, GRAPPA) |  | |
| Cardiac gating | Yes | **No** |
| If used, cardiac gating method:  (e.g. PPU or ECG) |  | |
| Contrast enhancement | Yes | **No** |
| If used, provide name of contrast agent, dose and timing of scan post-contrast administration |  | |

| Acquisition sequence | | |
| --- | --- | --- |
| Type  (e.g. FLAIR, DIR, DTI, fMRI) | SE T1 | |
| Acquisition time | 5.45 | |
| Orientation | Axial | |
| Alignment  (e.g. anterior commissure/poster commissure line) | Anterior commissure/posterior commissure | |
| Voxel size | 0.89 x 0.89 x 5.0 mm3 | |
| TR | 525 ms | |
| TE | 12 ms | |
| TI |  | |
| Flip angle | 90˚ | |
| NEX | 2 | |
| Field of view | 230 mm | |
| Matrix size | 256 × 256 | |
| Parallel imaging | Yes | **No** |
| If used, parallel imaging method:  (e.g. SENSE, GRAPPA) |  | |
| Cardiac gating | Yes | **No** |
| If used, cardiac gating method:  (e.g. PPU or ECG) |  | |
| Contrast enhancement | Yes | **No** |
| If used, provide name of contrast agent, dose and timing of scan post-contrast administration |  | |

| Image analysis methods and outputs | |
| --- | --- |
| ***Lesions*** | |
| Type  (e.g. Gd-enhancing, T2-hyperintense, T1-hypointense) | T2 hyperintense and T1 hypointense lesions |
| Analysis method | Semi-automated edge detection contouring/thresholding technique |
| Analysis software | JIM version 6.0 |
| Output measure  (e.g. count or volume [ml]) | mL |
| ***Tissue measures (e.g. MTR, DTI, T1-RT, T2-RT, T2*, T2’, ^1^H-MRS, perfusion, Na)*** | |
| Type  (e.g. whole brain, grey matter, white matter, spinal cord, normal-appearing grey matter or white matter) | Whole brain volume, Cortical volume |
| Analysis method | SIENAX |
| Analysis software | FSL |
| Output measure | mL |
| ***Tissue measures (e.g. MTR, DTI, T1-RT, T2-RT, T2*, T2’, ^1^H-MRS, perfusion, Na)*** | |
| Type  (e.g. whole brain, grey matter, white matter, spinal cord, normal-appearing grey matter or white matter) | Subcortical deep gray matter volume |
| Analysis method | FIRST |
| Analysis software | FSL |
| Output measure | mL |
